# Changes of thoracic duct flow and morphology in an animal model of elevated central venous pressure

**DOI:** 10.3389/fphys.2022.798284

**Published:** 2022-08-08

**Authors:** Xiao Lu, Mengjun Wang, Ling Han, Joshua Krieger, Jillian Ivers, Sean Chambers, Max Itkin, Daniel Burkhoff, Ghassan S. Kassab

**Affiliations:** ^1^ California Medical Innovations Institute, San Diego, CA, United States; ^2^ 3DT Holdings LLC, San Diego, CA, United States; ^3^ Cook Medical, Bloomington, IN, United States; ^4^ Center for Lymphatic Imaging and Interventions, University of Pennsylvania, Perelman School of Medicine, Philadelphia, PA, United States; ^5^ Cardiovascular Research Foundation, New York, NY, United States

**Keywords:** lymphatic flow, thoracic duct, central venous pressure elevation, tricuspid regurgitation, remodeling

## Abstract

**Objective:** Investigation of lymph fluid dynamics in thoracic duct during central venous pressure elevation.

**Background:** Lymphatic flow is affected by elevated central venous pressure (CVP) in congestive heart failure. The changes of thoracic duct (TD) lymph flow have not been studied chronically in the setting of elevated CVP. This study is to investigate fluid dynamics and remodeling of the TD in the elevated CVP animal model.

**Methods:** A flow probe was implanted on the swine TD (*n* = 6) and tricuspid regurgitation (TR) was created by cutting tricuspid chordae percutaneously. Six swine were used as control group animals. The TD flow was measured for 2 weeks (baseline) before TR and 4 weeks postop-TR surgery. Arterial pressure and CVP were measured. The pressure and flow in the TD were measured percutaneously. Histological and morphological analyses were performed.

**Results:** TR resulted in an increase in CVP from 4.2 ± 2.6 to 10.1 ± 4.3 mmHg (*p* < 0.05). The lymph flow in the TD increased from 0.78 ± 1.06 before TR to 8.8 ± 4.8 ml/min (*p* < 0.05) 2 days post-TR and remained plateau for 4 weeks, i.e., the TD flow remained approximately 8–11 fold its baseline. Compared to the 8.1 ± 3.2 mmHg control group, the TD average pressures at the lymphovenous junction increased to 14.6 ± 5.7 mmHg in the TR group (*p* < 0.05). The TD diameter and wall thickness increased from 3.35 ± 0.37 mm and 0.06 ± 0.01 mm in control to 4.32 ± 0.57 mm and 0.26 ± 0.02 mm (*p* < 0.05) in the TR group, respectively.

**Conclusion:** The elevated CVP results in a significant increase in TD flow and pressure which causes the TD’s outward remodeling and thickening. Our study implicates that the outward remodeling may result in the TD valve incompetence due to failure coaptation of leaflets.

## Introduction

The main function of the lymphatic circulation is to collect and transport interstitial fluid and extravasated plasma proteins, absorbed lipids, and other large molecules from the peripheral tissues back to the venous system primarily via the thoracic duct (TD) (Van Pernis, 1949; Kubik, 1973; Schmid-Schonbein, 1990; Michel, 1997; Yu et al., 2010; Moore and Bertram, 2018). The TD is the largest lymphatic vessel in the body and transports the majority of the lymph in the body except for the head, neck, arm, and right thorax in humans (Archimbaud et al., 1969; Kubik, 1973; Takahashi et al., 2003; Liu et al., 2006; Seeger et al., 2009). The lymphatic system is solely responsible for the removal of fluid from the tissue since there is no venous reabsorption of interstitial fluid (Schmid-Schonbein, 1990; Michel, 1997; Moore and Bertram, 2018). Elevated central venous pressure (CVP) in patients with congestive heart failure (CHF) results in increased fluid transudation from the capillary bed into the interstitial space, thus increasing the volumes of interstitial fluid to be removed by the lymphatic system (Dumont et al., 1963; Wegria et al., 1963; Cole et al., 1967; Szabo and Magyar, 1967; Witte et al., 1969; Kusaka et al., 2003; Aisenberg, 2013; Smart, 2017). Tissue edema in patients with CHF occurs when the overwhelmed lymphatic system fails to remove the fluid from the tissues (Dumont et al., 1963; Wegria et al., 1963; Szabo and Magyar, 1967; Kusaka et al., 2003; Aisenberg, 2013). The symptoms of circulatory congestion were dramatically relieved after venting the distended TD (Cole et al., 1967; Witte et al., 1969). Although the lymphatic system plays a significant role in the pathophysiology of the edema in CHF patients, the understanding of the fluidodynamic change of the lymphatic system in CHF is lacking.

All previous studies of lymphatic flow in humans as well as in animal models have been made under “open” conditions in which the TD had been externalized (i.e., catheterized and open to the atmosphere) when the flow was measured (Dumont et al., 1963; Wegria et al., 1963; Szabo and Magyar, 1967). However, elevated CVP not only increases the transudation of the fluid into tissues but also increases the hydraulic afterload on the lymphatic system, impeding propagation of the flow of the lymph into the venous system (Van Pernis, 1949; Yu et al., 2010; Seeger et al., 2009). For that reason, the measurement of the lymphatic flow draining in an external container (open to the atmosphere) does not allow to account for the impact of the increased hydraulic afterload. To the best of our knowledge, there have not been any chronic measurements of the TD flow in the intact lymphatic system most closely reflecting the clinical scenario.

Hence, the goal of this study was to record the changes in lymphatic dynamics using a chronically implantable flow probe on the TD in the setting of experimental heart failure with an elevated CVP. We used a tricuspid regurgitation (TR) swine model to elevate CVP. The TR model was created by selective disruption of the tricuspid valve chordae tendineae. The changes in lymphatic flow and pressure were monitored by a flow transducer that was chronically implanted on the TD and a pressure transducer in the TD that was placed by percutaneous catheterization, respectively. The structural and biomechanical aspects of the TD wall remodeling were characterized by chronic pressure and flow overload.

## Materials and methods

All animal experiments were performed in accordance with national and local ethical guidelines, including the Principles of Laboratory Animal Care, the Guide for the Care and Use of Laboratory Animals, and the National Society for Medical Research. The research protocol was approved by California Medical Innovations Institute IACUC. Twelve domestic pigs, weighing 55 ± 7 kg (51–63 kg), were obtained from a certified vendor and randomly assigned to two groups. The animals in the TR group (*n* = 6) were implanted with flow transducers on the TD and TR was obtained with an interventional procedure. The animals in the control group (*n* = 6) did not undergo TR and flow transducer implantation. The control animals were used for comparison of the TD pressure at the lymphovenous junction, as well as for postmortem heart mass and histological and morphometric analysis of the TD.

### Flow probe implanted on the TD

The animals in TR group (*n* = 6) were pre-anesthetized with Telazol (50 mg/ml), Ketamine (25 mg/ml), and Xylazine (25 mg/ml) and maintained with 2% isoflurane for all procedures. A 15 cm right lateral thoracotomy was performed between the 6th and 7th ribs. The ribs were separated with a thoracic retractor to create a 7–9 cm opening. The posterior mediastinum was exposed by lifting the right lung with a malleable retractor and the TD was identified alongside the aorta. The TD was then carefully dissected free from the aorta. A 1.5 mm diameter perivascular flow transducer with a 60 cm waterproof cable (1.5SB; Transonic Systems Inc.) was placed on the TD and sutured to the adjacent tissue to avoid movement. The cable of the flow probe was connected to Transonic Flowmeter (T420, Transonic Systems Inc.) using the extension cable to measure the TD flow. The thoracotomy was closed by suturing the muscle in layers (0-0 Gut chrome, Ethicon) including the subcutaneous connective tissue (3-0 PGA, Teleflex) and the subcutaneous skin (3-0 Proline, AD Surgical). Approximately 20 cm of the waterproof cable was placed within the thoracic cavity while the rest of the cable was located outside of the animal body and adhered to the dorsal skin. All animals were allowed to fully recover for a total of 2 weeks. The TD flow was measured by connecting the external cable to the Transonic flow meter via the extension cable while the animals ambulated in the cage. Measurements of the TD flow before TR surgery served as the baseline of subsequent time points after TR surgery.

### Percutaneous model of TR

**FIGURE 1:**
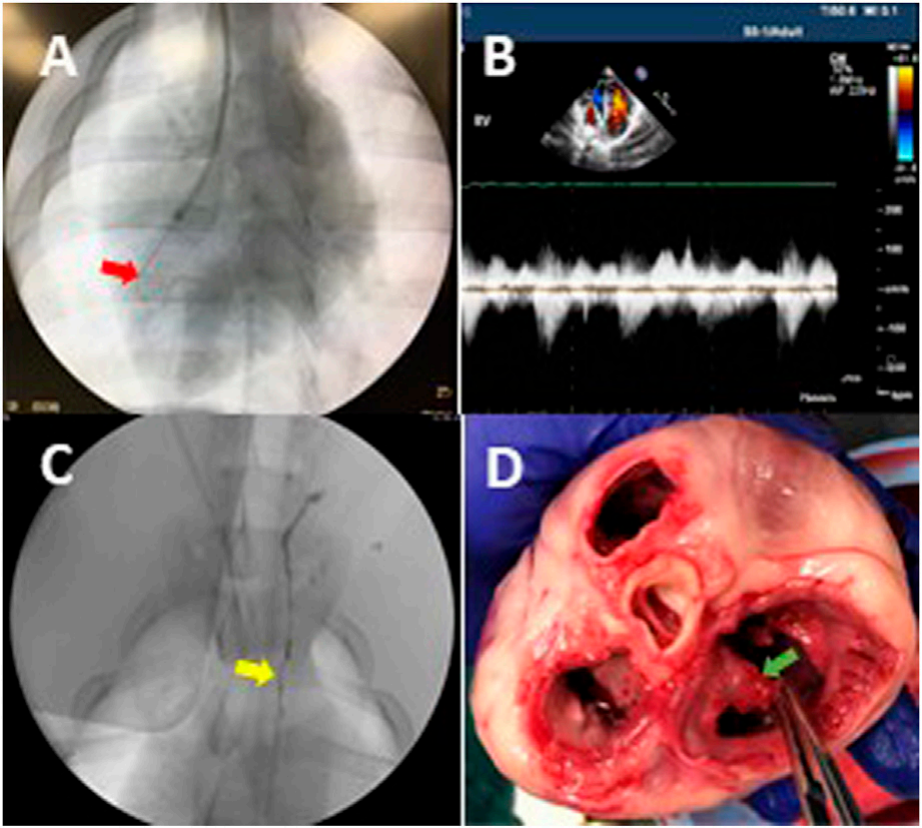
Animal model of tricuspid regurgitation (TR). **(A)** The fluoroscopic image represents that a cutting device was rotated to cut chordae tendineae in the right ventricle. The red arrow indicates the cutting device. **(B)** An ultrasonic image indicates the reflux in the right atrium (i.e., TR) after chordae disruption. **(C)** The TD was visualized during contrast injection. The yellow arrow indicates the TD. **(D)** An example of the cutting device caused chordae disruption and the zigzag edge of a leaflet in postmortem gross examination. The chordae of tricuspid leaflets were disrupted as desired (green arrow), which resulted in TR and elevated CVP.

After 2 weeks of flow probe implantation, the animals in the TR group (*n* = 6) were anesthetized, as described above. The animals were positioned on the fluoroscopy table, the neck was prepared in a sterile fashion and the jugular vein was accessed while artery pressure was monitored. An 8Fr vascular sheath was advanced from the jugular vein into the right atrioventricular junction under fluoroscopic guidance. A pressure transducer was connected to the side port of the sheath to monitor the jugular vein pressure (JVP), right atrium (RA) pressure (RAP), and right ventricle (RV) pressure (RVP). The CVP is approximately equal to JVP due to the jugular vein being near the central venous track. The guide catheter (Flexor^®^ Check-Flo^®^ 7Fr, Cook Medical) was advanced into the right ventricle through the sheath under fluoroscopic guidance. Through the guide catheter, a cutting device (Custom Cutting Device, Cook Medical) was introduced (Figure 1A). The chordae tendineae was then engaged by rotation of the cutting device toward the ventricular wall. The cutting device was then withdrawn slightly to check for engagement with the chordae. As soon as the chordal engagement was confirmed, the cutting device was retracted to cut the chordae. The chordae were cut one at a time until the pressure difference between the right ventricle and atrium decreased from ∼6 mmHg to less than 2 mmHg, indicating significant TR. The contrast was then injected into the right ventricle to demonstrate reflux into the right atrium. The vascular sheath was removed. The skin puncture was closed with a single suture for hemostasis. With the animal still under anesthesia, TR was further verified by cardiac echocardiography (Figure 1B). The animal was then recovered, returned to the cage, and allowed access to water and food. The animals were kept alive for 4 weeks postoperatively.

### TD flow measurement in conscious animals

The TD flow rate of each animal in the TR group was measured before TR surgery and on days 1, 2, 3, 7, 14, and 28 after TR surgery. The TD flow measurements before TR surgery served as the baseline. Each measurement was performed approximately 3 h after the last meal. The Transonic flowmeter with a 2 m extension cable was positioned outside of the cage to allow full movement within the cage. The external part of the flow probe cable was released from the swine jacket and connected to the extension cable of the flowmeter. Each time the TD flow was monitored for >10 min while the animal was free to move.

### TD-venous junction pressure measurements

On day 28, animals in the TR and the control groups were anesthetized and a 5Fr catheter was introduced into the femoral artery to monitor arterial pressure (AP) while another 5Fr catheter was introduced into the jugular vein to monitor venous pressure. Initially, the TD flow of animals in the experimental group was measured as described above. The bilateral inguinal lymph nodes were accessed with a 25G needle using ultrasonic (United States) guidance and the oil-based iodinated contrast agent (Lipiodol, Guerbet Laboratories) was injected into the nodes (Cope 1996; Itkin et al., 2010; Inoue et al., 2016). As soon as the lymphatic vessels and cisterna chyli (CC) were visualized under fluoroscopy, an afferent vessel of the CC was transabdominally accessed under fluoroscopic guidance using a 21G/20 cm needle (Chiba Biopsy Needle, Cook Medical) and then a 0.018″ guidewire (Hydrophilic Wire Guide, Cook Medical) was advanced in the CC through the needle for the TD catheterization with a microcatheter (2.8F Cantata microcatheter, Cook Medical) (Cope 1996; Itkin et al., 2010). Water-soluble iodinated contrast (Omnipaque, GE Healthcare) was injected through the microcatheter to visualize the TD via fluoroscopy (Figure 1C). A 0.014” guidewire with a pressure transducer (Primewire, Volcano) was advanced through the microcatheter into the TD and accessed the lymphovenous junction for pressure measurement.

### Gross examination of and ex vivo pressure-diameter testing of the TD

The right lateral thoracotomy between the 7th and 8th ribs was performed for visual examination in two groups. The fluid accumulation (edema) around the TD, the TD dilation, and lymph flow in the TD were assessed. The animals in both groups were then sacrificed. The heart and aorta with the TD were carefully excised and atria were excised from the heart. The injury of the tricuspid valve was visually examined. The ventricles were weighed.

A TD specimen (∼3 cm long) was isolated from the descending aorta. The TD specimen was submerged in a calcium-free physiological saline solution containing 2.5 mmol/L EGTA in a chamber. Both ends of the TD were cannulated with the cannulas which were connected to containers using Tygon tubes. The TD specimen was pressurized by raising a container that was connected to the proximal end of the TD specimen while the tube connected to a distal end of the TD specimen was occluded. The intraluminal pressure of the TD specimen was determined by the height of the container. The heights of the container were increased by 1 cm increments up to 15 cm and then to 20 and 30 cm. The images of the TD specimen were displayed on the screen with a CCD camera mounted on a stereo microscope and the diameter changes were measured with dimensional analysis software (DIAMTRAK 3+, Australia).

### Biomechanical analysis of the TD wall

The lymph wall shear stress (*WSS, τ*) and the TD circumferential wall stress (*CWS, σ*) were calculated to understand the biomechanical stimuli for the remodeling of the TD in TR. The lymph *WSS* of the TD was computed by the equation for laminar flow in a circular cylinder as:

$$\tau =\frac{32\mu Q}{\pi D_{i}^{3}}$$ (1)

where *D*_*i*_ is the internal diameter of the TD, *Q* is the volumetric lymph flow rate, and *μ* is the lymph viscosity.

The inner diameter of the TD in Eq. 1 was calculated from the incompressibility condition which, for a cylindrical vessel, can be expressed as:

$$D_{i}=\sqrt{D_{o}^{2}-\frac{4A_{o}}{\pi \lambda }}$$ (2)

where *D*_*i*_ and *D*_*o*_ are the inner and outer diameter of the TD at the loaded state, respectively, and *A*_*o*_ is the wall area in the no-load state which was measured from a transverse section of the TD. The stretch ratio in the axial direction, *λ* = l/l_o_, is also computed where l and l_o_ are the TD lengths in the loaded and unloaded state, respectively (i.e., the TD shortens axially when excised out). Hence, measurements of *D*_*o*_ *, λ,* and *A*_*o*_ allow the determination of *D*_*i*_ using Eq. 2 and *WSS* using Eq. 1.

The average *CWS* (*σ*) in the TD wall can be computed by cylindrical assumption as follow:

$$\sigma =\frac{P_{i}}{h/r}\;\;$$ (3)

where *P*_*i*_ is the TD pressure, and *r* is the TD inner radius. The lymph *WSS* and the TD *CWS* were determined at baseline and termination (28 days after initiation of TR), respectively.

### Histological and morphometric analysis of the TD

The TD specimens in both groups were harvested and fixed with 4% paraformaldehyde for histological measurements. The specimens were subsequently sectioned with a cryotome (CM 1850, Leica). The sections were processed with H&E (Hematoxylin and Eosin) staining. The microscopic evaluation was performed using a microscope (Eclipse E600, Nikon). The TD wall thickness was measured using ImageJ (NIH Image) software. The sections for immunofluorescence staining were blocked, permeabilized, and incubated with primary antibodies (anti-smooth muscle *α*-actin, ThermoFisher Scientific) overnight at 4°C. The sections were then incubated with fluorescence secondary antibodies (ThermoFisher Scientific) for 30 min. The fluorescence reagent (Life Technology) was used for cellular nuclei visualization. The images were obtained using a fluorescence microscope (Eclipse Ts2R, Nikon). The media and smooth muscle layer thicknesses were measured using ImageJ.

### Statistics

Data are presented as mean ± SD (standard deviation). Significant differences between groups were determined by Student’s *t*-test (two-tailed distribution, two-sample unequal variance). A probability of *p* < 0.05 was considered indicative of a statistically significant difference.

## Results

### CVP elevated in TR model

**FIGURE 2:**
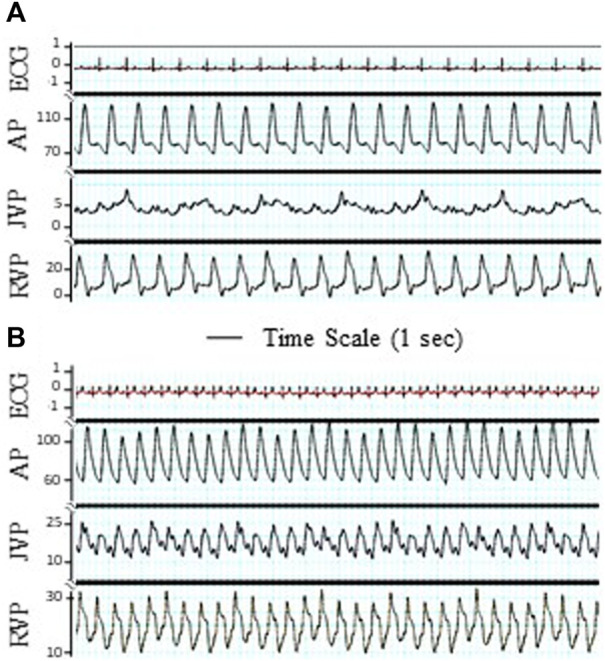
Tracings of electrocardiogram (ECG), artery pressure (AP), jugular vein pressure (JVP), and right ventricular pressure (RVP). The significant pulsation of JVP was observed after TR surgery. **(A)** Before TR. **(B)** After TR. ECG unit: mV. Unit of AP, JVP, and RVP: mmHg. Bar: Time scale for 1 s.

Postmortem gross examination showed that approximately 70% of the chordae tendinea of the tricuspid leaflets in each heart were disrupted (Figure 1D). The TR was verified by cardiac fluoroscopy, echocardiography, and a decrease in RV-to-RA pressure difference. The TR did not result in an increased heart rate, respiratory rate, arterial pressure, and RVP. The TR resulted in a significant increase in the systolic and mean pressures in the jugular vein and right atrium, which represent CVP (Table 1). The increase in systolic pressure in the jugular vein (Figure 2) was primarily due to the contraction of RV to compensate for TR. The RV masses statistically increased from 49.8 ± 8.6 in control to 87.1 ± 13.5 × 10^−3^ kg in TR group (*p* < 0.05). There was no statistically significant increase in the left ventricle (LV) mass in the TR group compared to controls, i.e., 154.1 ± 24.1 in control vs. 153.7 ± 18.9 g in the TR group (*p* > 0.05). There was a statistically increase in RV/LV ratio from 0.33 ± 0.04 in the control group to 0.56 ± 0.07 in the TR group (*p* < 0.05), providing evidence of significant RV hypertrophy.

**TABLE 1:** Pressures in the swine model of TR (unit: mmHg).

	Baseline		TR, post-op 10 min		TR, post-op 4 weeks	
	Systolic/Diastolic	Mean	Systolic/Diastolic	Mean	Systolic/Diastolic	Mean
Artery	107 ± 16/61 ± 11	73 ± 16	101 ± 19/60 ± 10	75 ± 13	97 ± 12/62 ± 8	74 ± 10
Jugular vein	7.4 ± 4.7/2.3 ± 2.1	4.2 ± 2.6	16.6 ± 5.5/2.2 ± 1.7^*^	8.4 ± 3.6	19.3 ± 5.4/3.7 ± 2.9^*^	10.1 ± 4.3
Right Atrium	8.8 ± 5.8/2.5 ± 2.7	5.6 ± 4.7	16.6 ± 5.1/4.2 ± 3.9^*^	9.8 ± 4.9	22.7 ± 7.5/3.7 ± 5.1^*^	12.0 ± 5.2
Right Ventricle	26.8 ± 5.7/1.8 ± 3.5	11.2 ± 5.7	20.2 ± 4.8/2.5 ± 3.3	10.8 ± 4.9	22.2 ± 7.6/4.8 ± 4.5	11.8 ± 5.1

Notes: TR: Tricuspid regurgitation (*n* = 6). *: *p* < 0.05 in comparison with control (*t*-test).

### TD flow measurement in conscious animals

**FIGURE 3:**
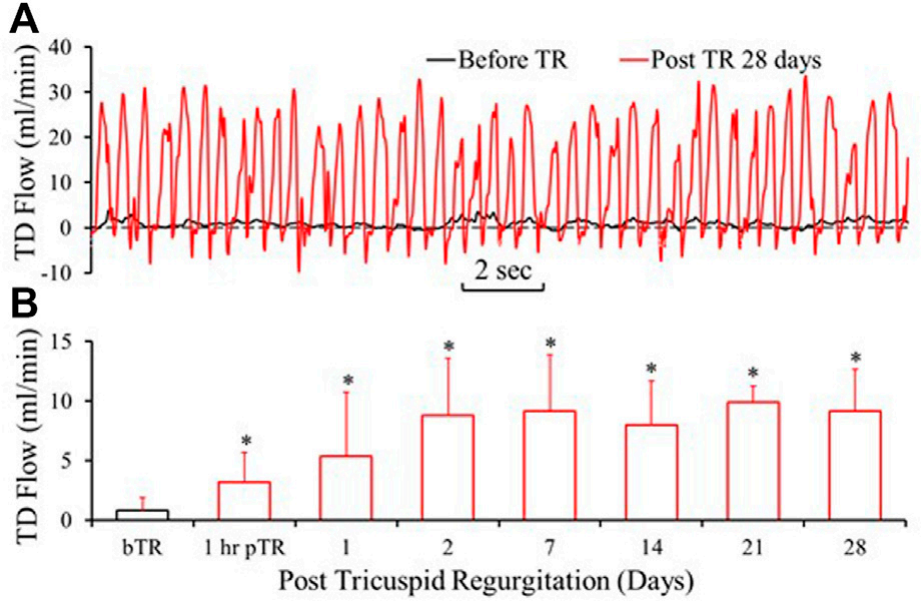
Lymph flow in the TD drastically increased after TR surgery. **(A)** Tracings of TD Lymph flow (TDQ) before TR surgery and 28 days post-TR surgery. **(B)** TDQ variation by the days of post TR surgery. bTR: Before TR surgery. 1 h pTR: 1 h post-TR surgery. *: *p* < 0.05 in comparison with bTR (before TR surgery).

The typical TD flow (TDQ) tracings at baseline (before TR surgery) and day 28 post TR surgery are represented in Figures 3A,B. The TDQ tracing on day 28 post-TR surgery showed increased pulsatile and average values compared to baseline (Figures 3A,B). Peak values of the TDQ waveforms on day 28 post-TR surgery varied randomly. The trough values of the TDQ waveforms showed substantial negative values (Figure 3B), indicating retrograde flow in the TD which was verified by direct visual observation of exposed segments of the TD during the terminal study (Supplementary Video S1) before catheterization of the TD (i.e., non-disturbance on the flow of the TD). The mean TDQ increased approximately 3-fold by 1 h after TR surgery induction and continued to increase, reaching the plateau of approximately 10-times baseline by day 2 post-TR surgery (Figure 3C). The mean TDQ increased from 0.78 ± 1.06 in baseline to 9.34 ± 3.54 ml/min (×1.67 × 10^−5^ L/s) on day 28 post TR surgery (Table 2).

**TABLE 2:** The changes of morphometric, lympho-dynamic, and biomechanical parameters of the TD due to TR.

	*D*_*o*_ (*mm*)	*D*_*i*_ (mm)	*h* (mm)	*TDQ* (ml/min)	*TDP* (mmHg)	*WSS* (dyn/cm^2^)	*σ*_*θ*_ (kPa)
Control, *n* = 6	3.35 ± 0.37	3.23 ± 0.29	0.06 ± 0.01	0.78 ± 1.06	8.2 ± 3.2	0.004 ± 0.005	19.5 ± 3.5
Day 28 postop TR, *n* = 6	4.32 ± 0.57^*^	3.81 ± 0.52^*^	0.26 ± 0.02^*^	9.34 ± 3.54^*^	14.6 ± 5.7^*^	0.032 ± 0.009^*^	10.3 ± 1.9^*^
Ratio (TR/control)	1.3 ± 0.14	1.2 ± 0.15	4.4 ± 0.52	11.7 ± 4.7	1.8 ± 0.5	8.4 ± 5.6	0.53 ± 0.11

Notes: TR: tricuspid regurgitation. *D*_*i*_: inner diameter at loaded state. *D*_*o*_: outer diameter at loaded state. *h*: wall thickness at loaded state. *TDQ*: flow rate in TD. *TDP*: transluminal pressure of the middle TD. *WSS*: fluid wall shear stress. *σ*_*θ*_: wall circumferential stress. *: *p* < 0.05 in comparison with control (*t*-test).

### TD-venous junction pressure

The pressure in the TD at the lymphovenous junction significantly increased from 8.2 ± 3.2 in control to 14.6 ± 5.7 mmHg (×133.32 Pa) in the TR group (*p* < 0.05) (Table 2). We also measured the pressures in CC which increased from 12.2 ± 4.3 in control to 18.9 ± 5.7 mmHg (×133.32 Pa) (*p* < 0.05).

### Pressure-diameter and biomechanical analysis

**FIGURE 4:**
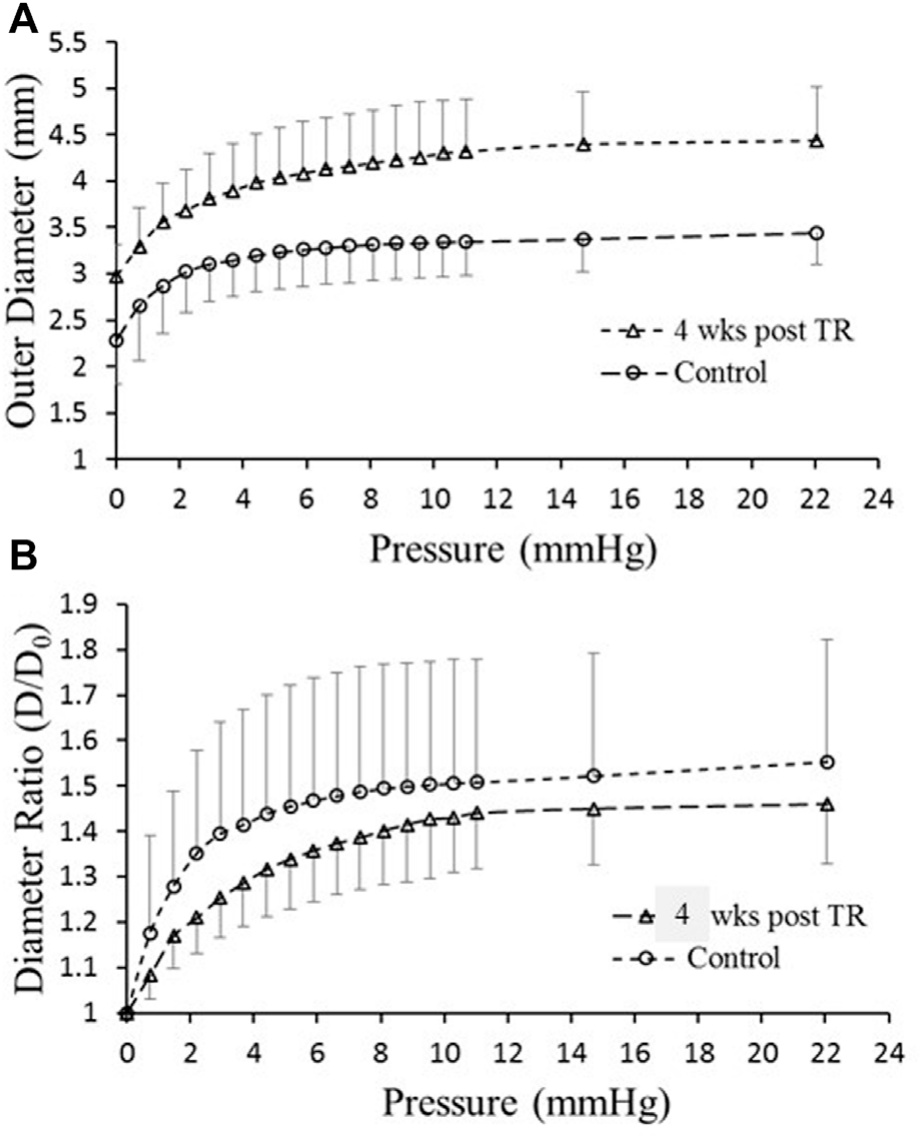
*Ex vivo* mechanical testing of TD. **(A)** Diameter versus pressure relationship for control and 28 days post-TR surgery. **(B)** Diameter ratio (degree of stretch) versus pressure for control and 28 days post-TR surgery.

The passive *in vitro* TD pressure–diameter relationship revealed that the diameter of TD increased (outward remodeling) significantly in the TR group compared to the control (Figure 4A). Although the diameter-to-wall thickness ratio, or stretch ratio, was slightly decreased in the TR group (Figure 4B), the change was not statistically significant compared to the control (*p* > 0.05). The morphometric, lymphodynamic, and biomechanical parameters measured are summarized in Table 2. These analyses show that lymph wall shear stress (*WSS*) was higher in the TR group than control due to the higher TD flow (Eq. 1; Table 2) in the TR group. The TD wall circumferential stress was lower in the TR group than the control due to the TD wall thickening (Table 2).

### Histological analysis of the TD

**FIGURE 5:**
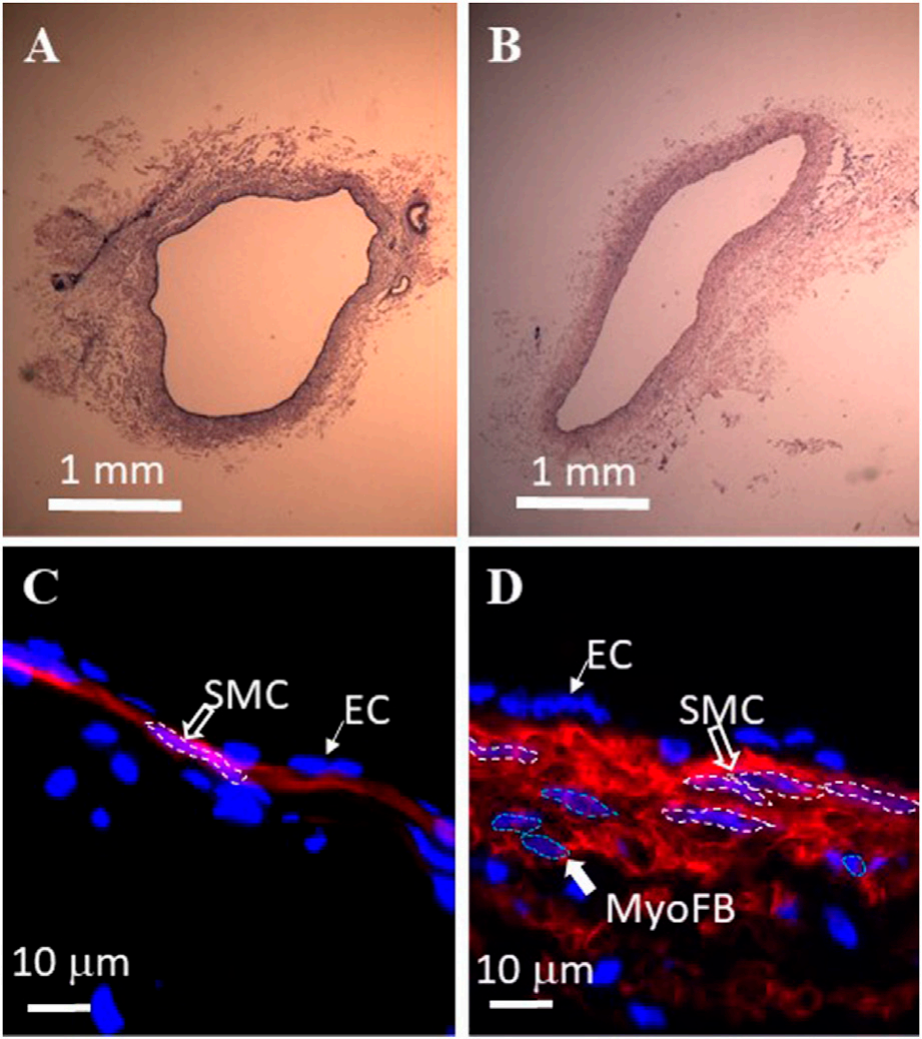
Histological analysis of swine thoracic duct for control and 28 days post-TR surgery. **(A)** Control in HE staining. **(B)** 28 days post-TR surgery in HE staining. **(C)** Immuno-florescence image of control. Red: α−actin. Blue: nuclei. SMC: smooth muscle cell. EC: endothelial cell. **(D)** Immuno-florescence image of 28 days post TR surgery. Red: α−actin. Blue: nuclei. MyoFB: myofibroblast.

The H&E staining of the TD in the control and TR group are represented in Figures 5A,B. There was a statistically significant increase in TD wall thickness (wall thickening) in the TR group in comparison control (Table 2). Smooth muscle hyperplasia was found in the media layer of the TD wall that increased from 7 ± 3 μm of control to 22 ± 11 μm in post-TR animals (*p* < 0.05). The smooth muscle layer increased from one-to-two layers in the control state to three-to-four layers in post-TR surgery animals (Figures 5C,D).

### Integration of results for summary and speculation

**FIGURE 6:**
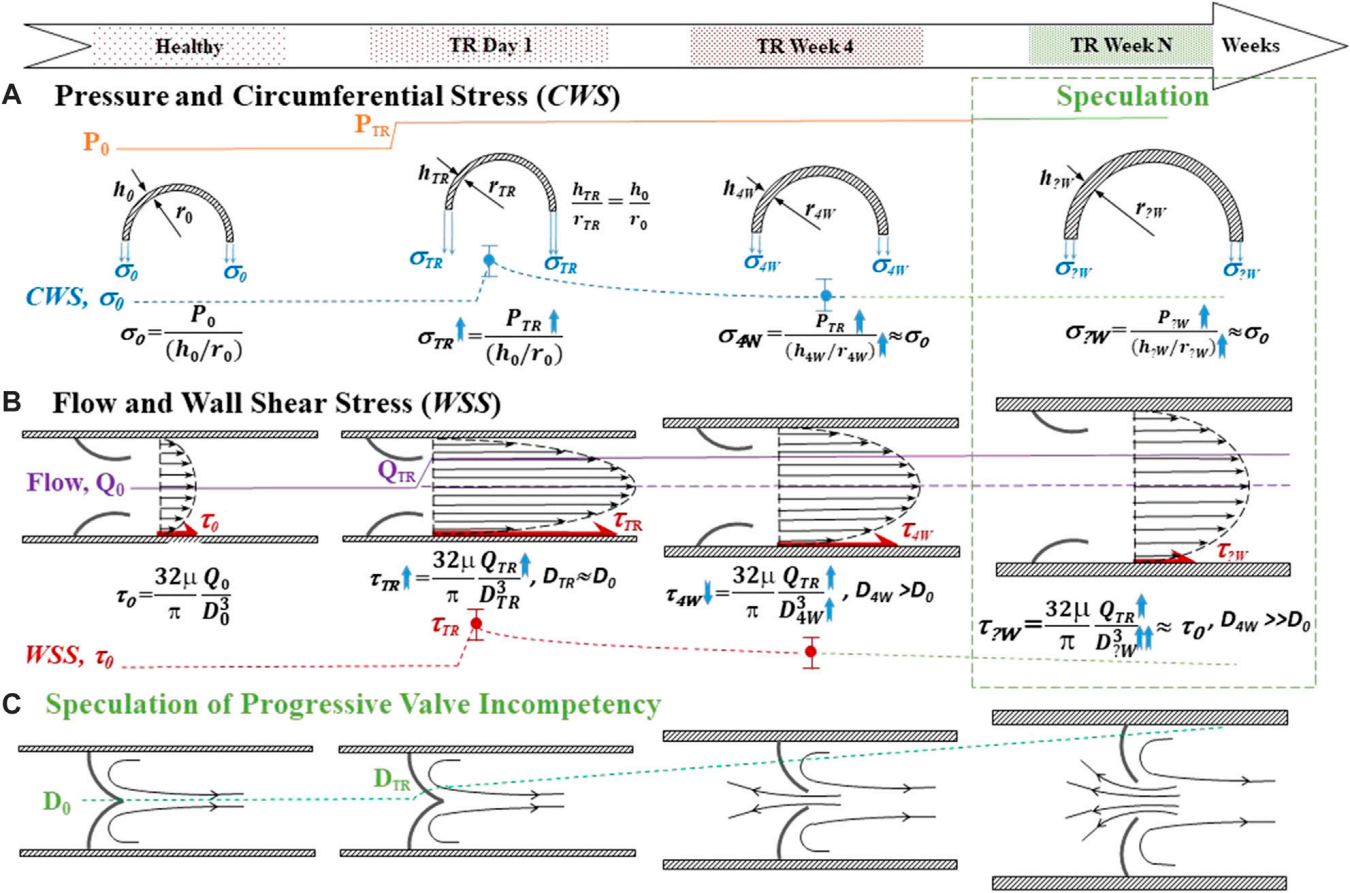
Schematic for integration of the fluidodynamic and geometric data of the TD and speculation of the long-term effect of post-TR surgery. Top panel: Time course. **(A)** Pressure and circumferential wall stress (*CWS*). The top represents the pressure rising in TR. The middle represents the transverse view of the TD when outward remodeling and wall thickening before, the onset of TR surgery, and 4 weeks post-TR surgery. The bottom represents the trend of *CWS* change with time course. **(B)** Flow rate and wall shear stress (*WSS*). The top represents the longitudinal view of the TD outward remodeling before, the onset of TR, and 4 weeks post-TR surgery. The bottom represents the trend of *WSS* change with time course. The column highlighted by the green box in Figures 6A,B represents our (logical) speculation on the remodeling and homeostasis of mechanical stresses exceeding 4 weeks post-TR surgery. **(C)** Speculation of progressive valve incompetency induced by outward remodeling: The longitudinal view of the outward remodeling of the TD results in TD valve malfunction. TR: tricuspid regurgitation. P: Pressure. h: Wall thickness of TD. r: Radius of TD. *CWS* (*σ*): Circumferential wall stress. Q: Lymph flow in TD. *WSS* (*τ*): Wall shear stress. D: Inner diameter of TD.

We present Figures 6A,B to integrate the fluid dynamic and geometric data of the TD to explain the effect of post-TR surgery on the fluid dynamics and morphometry of the TD. Figure 6A represents pressure, geometric parameters, and *CWS* before TR surgery, at the onset of TR surgery, and 4 weeks post-TR surgery (data from Table 2). Figure 6B represents flow rate, geometric parameters, and *WSS* before TR surgery, the onset of TR surgery, and 4 weeks post-TR surgery (data from Table 2). The column highlighted by the green box in Figures 6A,B represents our speculation on the remodeling and homeostasis of mechanical stresses exceeding 4 weeks post-TR surgery, i.e., the elevated pressure in the TD caused an overshoot of mechanical stresses (*CWS* and *WSS*) that gradually return to the level before TR surgery due to progressive outward remodeling (increase in diameter) and wall thickening.

## Discussion

In this study, we report the effect of elevated CVP in the setting of TR on lymph fluid dynamics. We observed that the TD lymph flow rate doubled nearly immediately following induction of TR and reached a plateau ∼10-fold that of baseline by approximately 2 days. The pressure in the TD increased proportionally to the elevated CVP. These lymphodynamic changes caused significant morphological remodeling of the TD which was characterized by increased TD diameter (outward remodeling), increased wall thickness (thickening), and hyperplasia of media smooth muscle layers.

The increase in TD lymph flow found in this study reflects an important pathophysiological consequence of elevated CVP in conditions such as congestive heart failure (CHF). The main function of the lymphatic system is to remove interstitial fluid from the tissues and return it to the venous circulation. Approximately 50 years ago, it was shown that there is no capillary venous reabsorption of interstitial fluid, revealing that the lymphatic system is solely responsible for the removal of interstitial fluid (Kubik, 1973; Schmid-Schonbein, 1990; Michel, 1997; Moore and Bertram, 2018). Elevations of CVP in CHF affect the lymphatic system in two ways: 1) increased lymph production due to increased plasma ultrafiltration from the capillary bed into interstitial tissue, especially in the liver portal circulation (Dumont et al., 1963; Wegria et al., 1963; Cole et al., 1967; Szabo and Magyar, 1967; Witte et al., 1969; Kusaka et al., 2003; Dib et al., 2006; Shimizu, 2008); and 2) increased TD afterload pressure at the lymphovenous junction which impedes TD flow. Based on conservation of mass principles, the rate of lymph accumulation equals the rate of lymph production minus the rate of lymph clearance by the lymphatic circulation. Hence, under normal physiological conditions, the rate of transudate production is equal to the rate of clearance. However, if the rate of production exceeds the rate of clearance, then the interstitial fluid is retained in the tissue resulting in tissue edema; if a new equilibrium condition cannot be reached, interstitial fluid accumulation can be progressive.

The effect of elevated CVP on TD flow has been an important topic of lymphatic research for over 135 years. Researchers previously reported that there is an exponential increase in the TD flow in relation to CVP in animal models and in patients with CHF (Dumont et al., 1963; Wegria et al., 1963; Szabo and Magyar, 1967). However, in all these clinical and experimental studies, the effect of elevated CVP was measured by externalization of the TD which creates an open system and hence eliminates the lymphatic flow impediment by increased venous pressures. The important question which has not been answered is whether there is an increase in TD lymph flow in a closed circuit in the setting of elevated CVP. In other words, which effect of elevated CVP is more important: increased TD afterload or increased lymphatic production? The dramatic, rapid, and sustained increase in TDQ following induction of severe TR indicates that increased lymph production is the dominant factor. This is further supported by the fact that the pressure difference across the TD (from the CC to the TD-venous junction) was not significantly altered following the induction of TR.

The diameter of the TD has been shown to increase in response to a rise in intra-TD pressure in conditions that result in an increased TD flow (Van Pernis, 1949; Takahashi et al., 2003; Liu et al., 2006; Seeger et al., 2009; Yu et al., 2010), including CHF, liver cirrhosis, and malignancy (Archimbaud et al., 1969; Takahashi et al., 2003; Seeger et al., 2009; Yu et al., 2010). In this study, the *ex vivo* pressure diameter (P–D) relationship shows that the TD diameter was enlarged 4 weeks post TR surgery relative to control. Although the enlargement of the TD may reduce the resistance to flow (Poiseuille’s law), it may cause insufficiency of the lymphatic valve leaflets due to loss of coaptation (Figure 6). Proper coaptation of the valve leaflets in response to negative pressure is necessary for unidirectional lymph transport and prevention of backflow (Koroleva, 1957; McHale and Roddie, 1975; Petrenko and Kruglov, 2004; Jamalian et al., 2016; Scallan et al., 2016). Therefore, malfunction of the TD valves may reduce the efficacy of the system to drain lymph and return it to the venous system. Indeed, the TD flow measured by the high-fidelity Transonic flow probe showed substantial negative flows (Figure 2C), suggesting retrograde flow and incompetence of the TD valves (Supplementary Video S1). Lymphatic valve insufficiency may cause a negative spiral of lymphatic failure where valve failure causes further volume-overload which further dilates TD and exacerbates valve function, and so on.

An increased rate of transudation of interstitial fluid can result in tissue edema and ascites in clinical settings. However, these were not observed in the present swine model of TR during the 4 weeks of study duration. We did observe significant fluid accumulation, however, around the TD (Supplementary Video S2), which implicates an increase in the TD filtration (lymph efflux across the TD wall). We hypothesize that the lack of overt signs of peripheral edema is because the right heart was still in a compensated state. Eventually, the increase in the TD filtration in the swine model of TR may result in more severe edema and ascites over time (Shimizu, 2008; Smart, 2017).

Vascular remodeling in response to hemodynamic forces (pressure and flow) is well established (McHale and Roddie, 1975; Kamiya and Togawa, 1980; Masuda et al., 1989; Petrenko and Kruglov, 2004; Hsu et al., 2011; Jamalian et al., 2016). It is well accepted that vessels remodel to restore shear and circumferential stress to the homeostasis of normal vessels (Figure 6) (Kamiya and Togawa, 1980; Masuda et al., 1989; Lu et al., 2001; Kassab et al., 2002; Hsu et al., 2011). More specifically, increased flow or wall shear stress induces diameter enlargement (outward remodeling) of arteries and veins (Kamiya and Togawa, 1980; Masuda et al., 1989; Lu et al., 2001; Hsu et al., 2011) whereas increased pressure results in significant arterial remodeling (wall thickening) to restore wall stress to homeostasis (Liu and Fung, 1996; Kassab et al., 2002). Based on our observations, the TD appears to follow a similar pattern of remodeling due to increased pressure and flow, i.e., flow-induced outward remodeling and pressure-driven thickening. The wall thickening offsets the elevation of the *CWS*. On the other hand, the outward remodeling counteracts the increase in *WSS*. The TD was exposed to both pressure and flow overload in the TR swine model (Figures 6A,B). The outward remodeling (increase in diameter) elevated the *CWS* (Eq. 3) and suppressed the *WSS* (Eq. 1), respectively. It is unclear whether the remodeling (outward and wall thickening) of the TD reestablishes the homeostasis for mechanical stresses. However, outward remodeling (increase in diameter) of the TD is deleterious for valvular leaflets coaptation because they do not grow/enlarge during outward remodeling. We speculate that the outward remodeling may result in lymphatic valve incompetency in the TD (Figure 6C), which compromises the lymph flow in the TD and leads to congestion in the TD.

### Study limitations

Although TR was immediately established by tricuspid chordae disruption in this study, the heart remained in a relatively compensated state. That is, the animal did not reach a state of decompensated CHF. Similarly, there were no obvious signs of fluid congestion or ascites which relates in part to the stage of heart failure. Longer durations are required to reach clinical signs of CHF. The implant surgery of the flow probe caused irreversible vasoconstriction and thereafter adjacent hyperplasia during the chronic period, which resulted in stenosis with ∼1.5 mm luminal diameter of the TD. The technical parameter of the invasive flow probe requires that the probe adapts on-site diameter of the TD (i.e., 1.5 mm) to obtain flow measurements. We did not observe a pressure gradient across the stenosis to be significantly different from other TD regions. We believe the TD flow may be underestimated in the TR swine model due to the surgical dissection of the TD for flow probe implant which results in some stenosis.

## Conclusion

To the best of our knowledge, this is the first study to chronically measure the TD flow with increased venous pressure in the setting of TR in a closed-loop circuit. We demonstrated an approximately 10-fold increase in lymph flow through the TD as well as increased pressure in the TD. These findings provide evidence that central venous pressure affects lymphatic production more than impedes lymphatic flow as the primary mechanism in the formation of interstitial edema. This increased flow results in the TD remodeling, i.e., increased TD diameter as well as increased wall thickness. The TD wall thickening offsets the increase in circumferential wall stress. However, the insufficient outward remodeling of the TD only partially counteracts the elevations of shear stress. Although the outward remodeling of the TD can eventually restore the wall shear stress levels, it can result in TD valve incompetence due to failure coaptation of valvular leaflets, as demonstrated by negative lymph flows. These findings provide important insights into the pathophysiology of interstitial congestion and may help guide the development of new therapies to treat symptoms in patients with elevated central venous pressures.

## Data Availability

The original contributions presented in the study are included in the article/Supplementary Material, further inquiries can be directed to the corresponding author.
