# Supplementary material for: Changes of thoracic duct flow and morphology in an animal model of elevated central venous pressure
Source: Front Physiol. 2022 Aug 8;13:798284. doi: 10.3389/fphys.2022.798284 (PMC9393243; doi:10.3389/fphys.2022.798284)
Supplement: Supplementary file 1 [file Table1.DOCX]

**Table 1.** Pressures in the swine model of TR (unit: mmHg).

|  | Baseline | | TR, post-op 10 min | | TR, post-op 4 week | |
| --- | --- | --- | --- | --- | --- | --- |
|  | Systolic/Diastolic | Mean | Systolic/Diastolic | Mean | Systolic/Diastolic | Mean |
| Artery | 107±16/61±11 | 73±16 | 101±19/60±10 | 75±13 | 97±12/62±8 | 74±10 |
| Jugular vein | 7.4±4.7/2.3±2.1 | 4.2±2.6 | 16.6±5.5/2.2±1.7^*^ | 8.4±3.6 | 19.3±5.4/3.7±2.9^*^ | 10.1±4.3 |
| Right Atrium | 8.8±5.8/2.5±2.7 | 5.6±4.7 | 16.6±5.1/4.2±3.9^*^ | 9.8±4.9 | 22.7±7.5/3.7±5.1^*^ | 12.0±5.2 |
| Right Ventricle | 26.8±5.7/1.8±3.5 | 11.2±5.7 | 20.2±4.8/2.5±3.3 | 10.8±4.9 | 22.2±7.6/4.8±4.5 | 11.8±5.1 |

Notes: TR: Tricuspid regurgitation (*n*=6). *: p<0.05 in comparison with control (*t*-test).
