# Supplementary material for: Changes of thoracic duct flow and morphology in an animal model of elevated central venous pressure
Source: Front Physiol. 2022 Aug 8;13:798284. doi: 10.3389/fphys.2022.798284 (PMC9393243; doi:10.3389/fphys.2022.798284)
Supplement: Supplementary file 2 [file Table2.DOCX]

**Table 2.** The changes of morphometric, lympho-dynamic, and biomechanical parameters of the TD due to TR.

|  | *D_o_*  *(mm)* | *D_i_*  (mm) | *h*  (mm) | *TDQ*  (ml/min) | *TDP*  (mmHg) | *WSS* (dyn/cm^2^) | *σ_ _*  (kPa) |
| --- | --- | --- | --- | --- | --- | --- | --- |
| Control, *n*=6 | 3.35±0.37 | 3.23±0.29 | 0.06±0.01 | 0.78±1.06 | 8.2±3.2 | 0.004±0.005 | 19.5±3.5 |
| Day 28 postop TR, *n*=6 | 4.32±0.57^*^ | 3.81±0.52^*^ | 0.26±0.02^*^ | 9.34±3.54^*^ | 14.6±5.7^*^ | 0.032±0.009^*^ | 10.3±1.9^*^ |
| Ratio (TR/control) | 1.3±0.14 | 1.2±0.15 | 4.4±0.52 | 11.7±4.7 | 1.8±0.5 | 8.4±5.6 | 0.53±0.11 |

Notes: TR: tricuspid regurgitation. *D_i_*: inner diameter at loaded state. *D_o_*: outer diameter at loaded state. *h*: wall thickness at loaded state. *TDQ*: flow rate in TD. *TDP*: transluminal pressure of the middle TD. *WSS*: fluid wall shear stress. *σ_θ_*: wall circumferential stress. *: p<0.05 in comparison with control (*t*-test).
